# Ultra-Wideband Magneto-Optic B-Field Sensor

**DOI:** 10.3390/s26154755

**Published:** 2026-07-27

**Authors:** Dong Ho Wu, Aaron M. Dougherty

**Affiliations:** Naval Research Laboratory, Washington, DC 20375, USA; aaron.m.dougherty.civ@us.navy.mil

**Keywords:** magneto-optic materials, ultra-wideband, extreme dynamic range, high sensitivity, high and low field sensing capability, non-intrusive field sensing, room-temperature operation, compact sensor, vector field sensing, magnetic domains

## Abstract

Among the various types of magnetic field sensors, only a few can detect magnetic fields non-intrusively, and even fewer offer ultra-wideband capability with an extreme dynamic range. In this report, we present a magneto-optic B-field probe that can detect a magnetic field with negligible perturbation to the field it detects. The probe is compact and has a frequency bandwidth of DC to 2 GHz. Furthermore, this technique achieves a dynamic range that significantly exceeds 100 dB by utilizing different magneto-optic materials. The device is an intrinsic vector-field sensor that can measure the direction, amplitude, and phase of a time-varying magnetic field. It offers several advantages, including a compact size, high sensitivity, room-temperature operation, a wide dynamic range, and vector-sensing capabilities across a broad frequency range. These features make it suitable for a variety of applications, such as RF and microwave testing, extreme low-frequency applications, and biomedical uses.

## 1. Introduction

There are many types of magnetic field sensors designed for specific applications. For instance, weak magnetic fields, ranging from femto-Tesla to micro-Tesla, can be accurately measured using a superconducting quantum interference device (SQUID) or an atomic vapor cell magnetometer, which operates effectively for fields ranging from 0.1 pico-Tesla to micro-Tesla. Other sensor technologies, commonly used in industrial settings, rely on various principles, including magneto-refractive materials, the magneto-optic effect, the Hall effect, optical fiber techniques, flux gate methods, magneto-resistive (MR) methods, and giant magnetoimpedance (GMI) techniques, as detailed in references [1,2,3,4,5,6,7,8].

Many of them contain metallic or semiconductor components that perturb the magnetic field they measure. In other words, they are invasive. These sensor technologies generally operate over a limited dynamic range, which can range from a few femto-Tesla to hundreds nano-Tesla, pico-Tesla to tens of milli-Tesla, or hundreds nano-Tesla to nearly one Tesla. However, none of these technologies can encompass the entire dynamic range from pico-Tesla to hundreds of Tesla. Furthermore, the operating frequency of these sensors is generally below 1 MHz and rarely exceeds 1 GHz. Most of them measure only the field’s strength, lacking the capability to measure vector fields.

In this paper, we present a magneto-optic (MO) magnetic field sensor technology developed at the Naval Research Laboratory (NRL). This technology is based on the Faraday effect in conjunction with an ellipsometry technique that utilizes magneto-optic materials. The prototype sensors are compact, typically measuring between 2 and 5 cm in length and 3 to 5 mm in diameter. They have a frequency bandwidth ranging from DC to 2 GHz. These sensors can detect magnetic fields as low as 1 pico-Tesla and as high as 100 to 120 Tesla, depending on the type of MO material used and with some minor modifications to the sensor’s design.

The sensor is inherently a vector sensor, capable of measuring the direction, amplitude, and phase of a time-varying magnetic field. Additionally, the sensor negligibly interferes with the magnetic field it measures, allowing multiple sensors to be placed close to one another. By taking advantage of this noninvasive nature and the sensor’s vector sensing capability, a three-axis magnetic field sensor can be created by arranging three MO sensors orthogonally along the x, y, and z axes.

With the appropriate MO material, an MO field sensor can accurately measure only the magnetic field and remains unaffected by the electric field, even in environments where both fields are present. Similarly, an electro-optic (EO) field sensor, the counterpart to the MO field probe, responds exclusively to the electric field [9]. By utilizing both MO and EO field sensors, it is possible to directly measure the Poynting vector S = E × H, which represents the directional energy flux—essentially the energy transfer per unit area per unit time, or the power flow of an electromagnetic field. Additionally, one can determine the impedance, Z = E/H, where E and H denote the electric and magnetic fields, respectively. Measuring these quantities can be quite challenging, particularly in inhomogeneous media. However, such measurements can be easily conducted using the MO and EO field sensors reported here and in reference [9].

**Figure 1:**
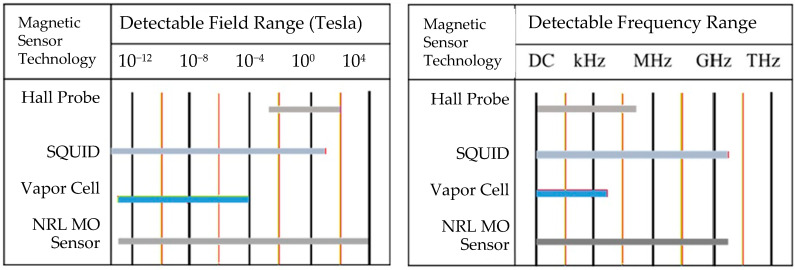
Detectable field ranges and frequency ranges that some magnetometer technologies can achieve. For example, measuring magnetic fields over a wide dynamic range can be achieved using various types of SQUIDs. Both the atomic vapor cell magnetometer and the NRL magneto-optic field probe exhibit high sensitivity and can detect vector fields [10].

Of various magnetometers, SQUIDs and atomic vapor cells provide the highest sensitivity, and Hall probes may be commonly found for various applications. We compare our MO field sensor with these well-known magnetometers in Figure 1 and Table 1, which show a typical field range, frequency range, sensor size, operating temperature and whether the probe interferes with the field that it measures.

## 2. Magneto-Optic Principles

**Figure 2:**
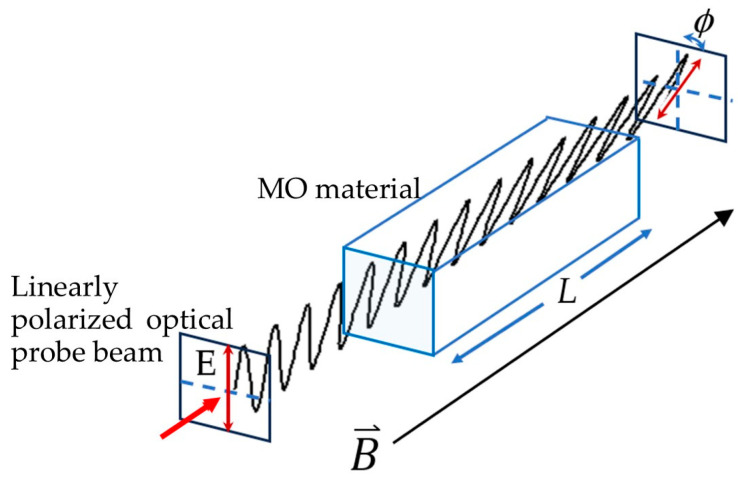
An illustration to show the Faraday effect, which results in the rotation of the polarization of a linearly polarized light beam, often referred to as the probe beam. In this figure, the vertical red arrow labeled “E” on the front plate and the red arrow tilted at an angle ϕ on the end plate represent the optical field of the linearly polarized probe beam, while $\overset{⃑}{B}$ indicates the external magnetic field, which is applied parallel to the probe beam as it travels through an MO material.

The magneto-optic B-field probe we report here is based on the Faraday effect discovered by Michael Faraday in 1845. As shown in Figure 2, when a linearly polarized light beam, which is typically a laser beam, passes through a magneto-optic material to which an external magnetic field is applied along the length of the material, the polarization of the beam rotates in proportion to the magnetic field strength. The polarization rotation angle (ϕ) can be expressed as

(1) $$\phi =VL\overset{⃑}{B}\cdot \hat{z}$$

where V, L, $\overset{⃑}{B}$, and $\hat{z}$ are the Verdet constant, the MO material’s length, the B-field vector, and the unit vector of the z-direction, which is parallel to the length of the MO material and aligns with the direction of the probe beam. The Verdet constant depends on the type of MO material and on the wavelength of the laser beam [11,12].

**Figure 3:**
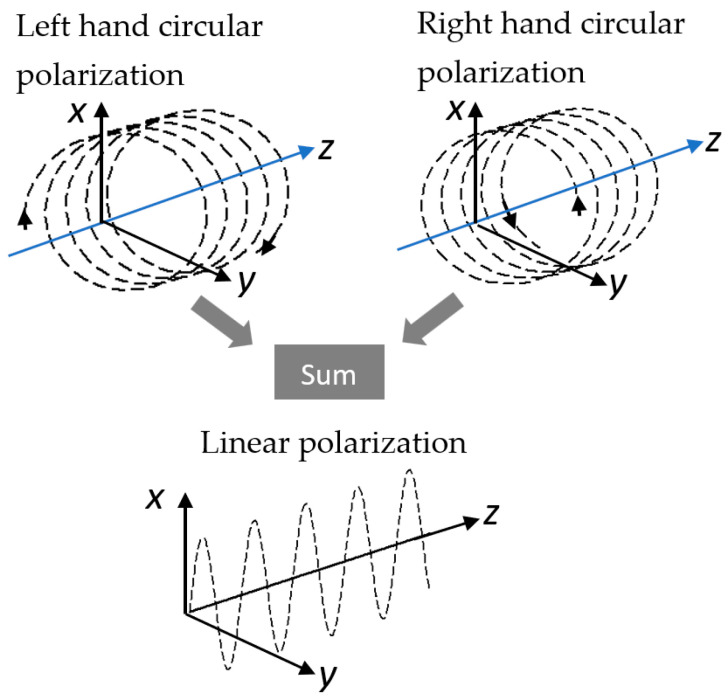
A linearly polarized light beam can be viewed as a combination of equal amounts of left-hand circularly polarized photons and right-hand circularly polarized photons.

When a magneto-optic material is exposed to a light beam with photon energy of *ħ*ω, the electrons in the material transition from the ground state to an excited state. Then, subsequently, the excited electrons emit photons and transition back to the ground state. A linearly polarized beam, which can be viewed as a combination of equal amounts of left-hand circularly polarized photons and right-hand circularly polarized photons, as illustrated in Figure 3, enters and exits the magneto-optic (MO) material without any polarization rotation if no magnetic field is applied to the material.

**Figure 4:**
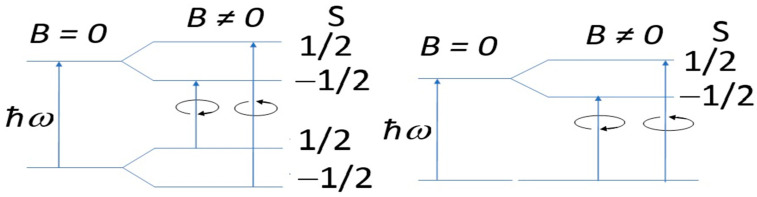
The excitation of electrons in paramagnetic or diamagnetic material with and without the Zeeman splitting. The circular arrows indicate the transitions for left-hand and right-hand circular polarization of photons.

However, when the material is subjected to a magnetic field, the ground and excited states split into two distinct energy levels corresponding to spin −1/2 and spin +1/2, as shown in Figure 4. This phenomenon is known as the Zeeman splitting. Due to the selection rule, which is based on the conservation of angular momentum, electrons can transition from the ground spin +1/2 state to the excited spin −1/2 state, which is associated with left-hand circular polarization, or from the ground spin −1/2 state to the excited spin +1/2 state, which corresponds to right-hand circular polarization, as illustrated in Figure 4.

When magnetic atoms or molecules become magnetized, the populations of the two spin states differ. As a result, the absorption of one circularly polarized light will be greater than that of the other. This results in a disparity between the populations of right-hand and left-hand circularly polarized light, which leads to the polarization rotation of the light beam. The above picture for the magneto-optic Faraday effect seems to be simple, and utilizing the Faraday effect for a magneto-optic sensor seems straightforward. However, the magneto-optic effect in magnetic materials is quite complex, and so is the realization of MO sensors.

## 3. Magneto-Optic Materials and Magnetic Behavior

We intended to develop a nonintrusive B-field sensor for radio frequency (RF) and microwave applications, targeting a dynamic range of over 55 dB and a frequency bandwidth spanning the entire RF and microwave spectrum. To achieve this, we searched for MO materials with a large Verdet constant and a wide bandwidth. We explored the CdMnTe and Bi:RIG families of materials. Although the CdMnTe family can display a significant Verdet constant and has the potential to detect a wide dynamic range of magnetic fields, it also possesses a weak electro-optical response that does not fulfill our requirements. We needed MO material that responds exclusively to a magnetic field. Therefore, we decided to focus solely on the Bi:RIG family, specifically the variant with the chemical composition of $\left\{{Bi}_{x}{Gd}_{3-x-y}{Lu}_{y}\right\}[{Fe}_{2-z_{a}}{Ga}_{z_{a}}]({Fe}_{3-z_{d}}{Ga}_{z_{d}})O_{12}$. The Bi:RIG materials are in thick-film form, and their magnetic domain structures and MO properties vary significantly by changing the chemical contents (e.g., *x*, *y*, *z_a_* and *z_d_*) [9]. They were initially developed for several applications, such as optical switches, optical circulators, and magneto-optical modulation within laser resonators [13,14].

The thick films exhibited ferrimagnetic properties, resembling ferromagnetic materials and displaying a spontaneous magnetic moment below the Curie temperature. Their intricate crystal structures lead to a combination of parallel and antiparallel alignments among neighboring atoms. However, because the opposing spins have unequal magnetic moments, the net magnetization is lower than that of typical ferromagnetic materials.

**Figure 5:**
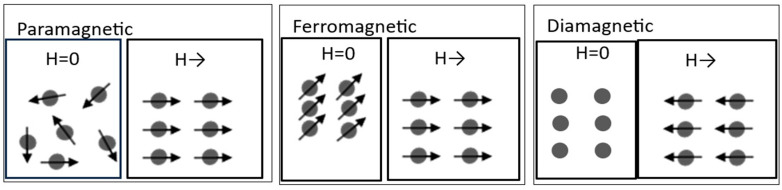
The spin states of atoms in paramagnetic, ferromagnetic, and diamagnetic materials with and without a magnetic field. The arrow next to H shows the direction of the magnetic field, while the arrows within the circular dots represent the spin states of the atoms.

Figure 5 illustrates the spin state of atoms in paramagnetic, ferromagnetic, and diamagnetic materials. The ferromagnetic materials typically exhibit magnetic domains at temperatures below Curie temperature. Similarly, our MO materials that are ferrimagnetic tend to have magnetic domains.

In ferromagnetic or ferrimagnetic materials, such as our MO thick films, the net spin state is nonzero even in the absence of an external magnetic field. In those materials, the Zeeman splitting is established during the crystal growth and is typically considered to remain constant [15]. This means the Zeeman splitting is chemically bound. As a result, the magneto-optic polarization rotation ($\theta_{F}$) primarily arises from the dot product between the crystal’s magnetization vector and the probe beam propagation path (i.e., the k-vector of the probe beam) through the crystal length L, as shown in Equation (2).

(2) $$\theta_{F}=L\alpha_{K}(\hat{k}\cdot M)$$

This equation resembles Equation (1), but it replaces the B-field vector $\overset{⃑}{B}$ and the Verdet constant V with the magnetization vector M and the Kundt’s constant $\alpha_{K}$. Now Kundt’s constant $\alpha_{K}$ relates the magnetic field strength to the polarization rotation [16]. For each material, $\alpha_{K}$ depends on the wavelength of the light and the temperature. In ferrimagnetic garnets, it is angular momentum conservation between the spin 1 photons and the unpaired spin 1/2 electrons that determine whether an excitation will take place, where the unpaired spin 1/2 electrons make up the crystal’s magnetization vector. Consequently, the chemically bound Zeeman effect shows a preference for the excitation of one electron spin state over the other, resulting in a phase retardation in either the right or left circular polarization components of the probe beam that leads to the polarization rotation of the beam.

As our MO thick films are ferrimagnetic, they tend to form magnetic domains in which the magnetic moments are aligned within the material. These magnetic domains can change when they are exposed to an external magnetic field, both the alignment of the domains and the boundaries between different domains. The former is called the domain motion, and the latter is the domain wall motion. In a magnetic material, there is a preferred direction of magnetization, which is called the easy axis. So, the magnetic-field response (both speed and amplitude) of the domain motion and the wall motion depend on the direction of the external field.

Our MO field sensor measures magnetic field strength by detecting the polarization rotation angle of a probe beam passing through a small sampling area of the magnetized MO material. It is ideal if the sampling area is within a single, homogeneously magnetized magnetic domain, and the magnetic domain area does not change with the external field strength. Under these ideal conditions, the design and operation of the MO field sensor are straightforward. However, generally, the diameter of a probe beam is larger, often measuring a few hundred micrometers, compared to the typical width of a magnetic domain, which ranges from 1 to 100 micrometers. Also, the magnetic domain area can change when it is exposed to an external magnetic field.

In addition, because the MO material we use is thick films with a typical thickness of less than 500 micrometers, we had to stack many of them to increase the length *L* so that we could achieve a larger polarization rotation, meaning a higher sensitivity, as one can see from Equation (2). As illustrated in Figure 10, the probe beam traverses multiple stacked magneto-optical (MO) films, each of which may display unique magnetic domain patterns and magnetic moments. As a result, the polarization rotation of the probe beam represents an averaged magneto-optical effect, which arises from interactions with various magnetic domains.

When subjected to an external magnetic field, magnetic domains and domain walls either change domain direction or domain size (either expanding or contracting), or the field may change both domain direction and domain size simultaneously. In other words, Kundt’s constant $\alpha_{K}$ is a combination of two different parameters: $\alpha_{W}$ related to domain wall motion and $\alpha_{D}$ related to domain motion, as shown in Equation (3) [17].

(3) $$\alpha_{K}={\eta \left(\phi_{i}\right)\alpha }_{D}+{\zeta \left(\phi_{i}\right)\alpha }_{W}$$

Here, $\eta \left(\phi_{i}\right)$ and $\zeta \left(\phi_{i}\right)$ are parameters that depend on the direction of the probe beam and of the external magnetic field applied to the MO material.

In the MO material, domain wall motion occurs along the easy axis, the direction in which a magnetic material can be magnetized with minimal energy, while domain rotation occurs towards the hard axis, the direction requiring more energy to magnetize the material than the easy axis. In other words, the MO responses related to $\alpha_{W}$ and $\alpha_{D}$ can be noticeably different depending on the direction of an external magnetic field applied to the MO material.

**Figure 6:**
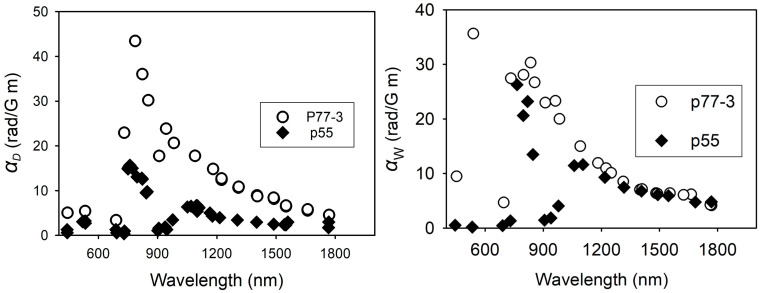
Probe beam’s wavelength dependence of Kundt’s constants $\alpha_{D}$ and $\alpha_{W}$, corresponding to domain motion and domain wall motion, respectively.

We also suspected that Kundt’s constants $\alpha_{W}$ and $\alpha_{D}$ may depend on the wavelength of the probe beam. Before examining the field-directional dependence of the MO response in the thick films, we measured the Kundt constants for several thick films grown under different conditions to determine whether domain wall motion plays a more dominant role in the MO response than domain motion. In Figure 6, we present data for two representative thick film samples, P77-3 and P55.

The data illustrate how the wavelength of the probe beam affects Kundt’s constants, $\alpha_{D}$ and $\alpha_{W}$. Both $\alpha_{W}$ and $\alpha_{D}$, corresponding to the MO responses related to domain wall and domain motions in these samples, are inversely proportional to the beam wavelength when the wavelength exceeds 700 nm. They exhibit a maximum at wavelengths around 700–750 nm, indicating that this range may be the optimal probe-beam wavelength for achieving the greatest Faraday rotation in the MO materials.

Interestingly, $\alpha_{D}$ in P55 is noticeably lower than that of P77 while $\alpha_{W}$ of P55 is nearly comparable to that of P77. The data indicate that in P55, the Faraday rotation is largely influenced by the domain wall motion, which is slow. In contrast, for P77-3, the domain motion has a slightly greater contribution to the Faraday rotation than the domain wall motion. Based on these data, we can predict that the magneto-optical (MO) response of P55 will be significantly influenced by the direction of the external magnetic field, while the response of P77 will show less dependence on the direction of the magnetic field.

**Figure 7:**
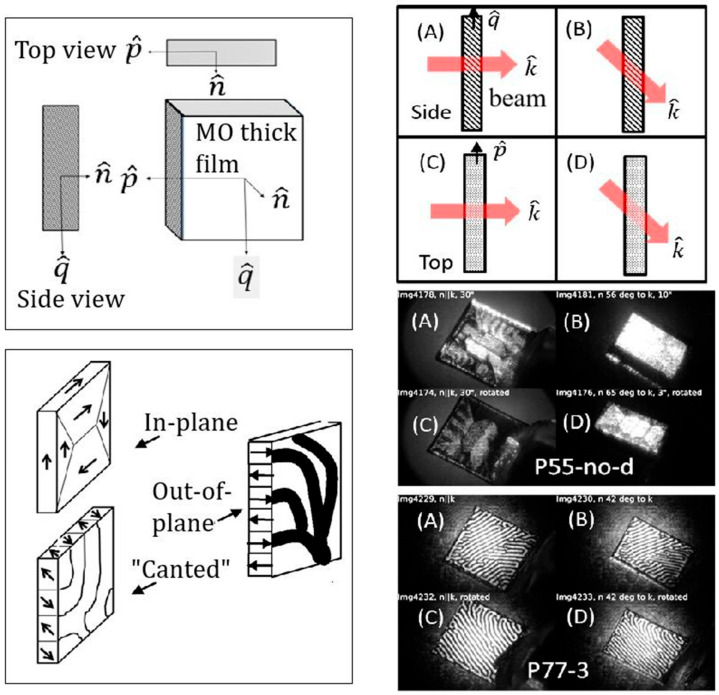
Magnetic domain patterns of our MO thick-film samples were examined using the polarized light microscopy. A polarized light beam was incident on the samples at various angles, as illustrated in the top right panel which depicts four different directions of the polarized beam during the polarization microscopy. We used four configurations: (A), (B), (C), and (D), based on the sample position and the probe beam direction. The top-left panel defines the reference axes: $\hat{n}$, $\hat{p}$, and $\hat{q}$, which are normal to the surface of the MO thick film, to the side surface, and to the top surface of the thick film, respectively. The bottom left panel illustrates an in-plane magnetic domain, an out-of-plane magnetic domain, and a canted magnetic domain. The bottom-right panel shows polarization microscopy images of samples P55 and P77-3, obtained with four different probe-beam configurations (A), (B), (C), and (D).

Next, we analyzed the magnetic domain patterns of our thick-film samples using polarized light microscopy. This examination involved irradiating a polarized light beam in four different configurations, as shown in the top-right panel of Figure 7, with the reference axes of the thin film illustrated in the top-left panel. The results are presented in the bottom-right panel of Figure 7. The sample P55 exhibited larger in-plane magnetic domains with fewer domain boundaries, resembling a pattern in reference [18], while P77-3 displayed narrow, intricately shaped meander-line domains. 

**Figure 8:**
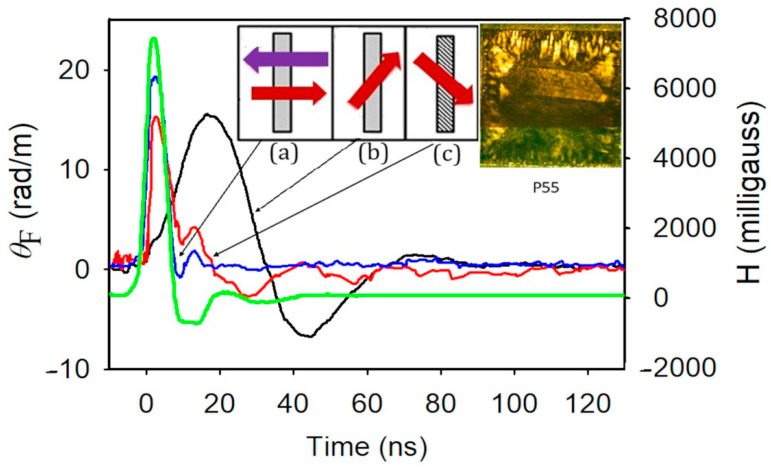
The MO response was measured with different probe beam directions while the magnetic pulse direction was set perpendicular to the MO thick-film sample, P55. The scale on the right indicates the strength of the external magnetic pulse. Sample P55 demonstrates larger magnetic domains with fewer domain walls, as shown in the inset polarization microscopy image. All waveforms were recorded using a digital oscilloscope. When the magnetic pulse and the probe beam are perpendicular to the surface of the MO thick film (illustration (**a**) in the inset), the MO signal (blue waveform) closely resembles the waveform of the external magnetic pulse (green waveform). Each black arrow shows the corresponding waveform for each configuration: (**a**) corresponds to the blue waveform, (**b**) to the black waveform, (**c**) to the red waveform.

**Figure 9:**
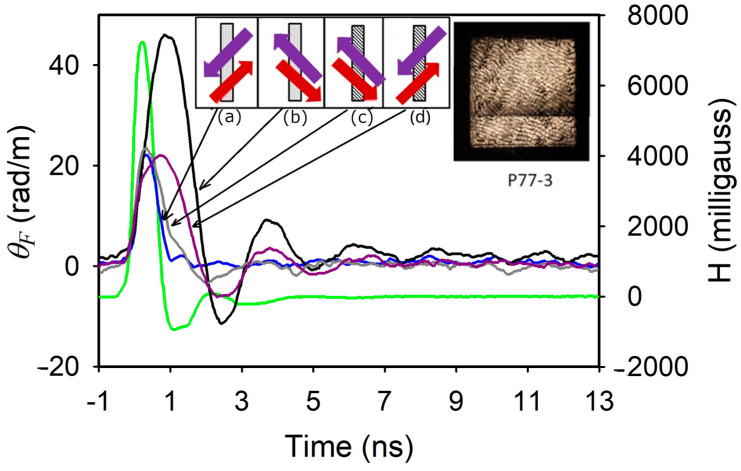
The MO polarization rotation response for the thick film sample P77-3, which has narrow, complex meander-line magnetic domains, as shown in the inset polarization microscopy image. The MO response varies significantly depending on the direction of the magnetic field and the probe beam. The black arrow shows the corresponding waveform for each configuration: (**a**–**d**). The green waveform represents the external magnetic pulse.

We studied the impact of domain patterns on the magneto-optic response by measuring the polarization rotation of a probe beam as we varied the directions of the magnetic fields and the probe beam across several thick-film samples grown under various conditions. The experiments revealed a range of distinct magneto-optic responses. Here, we focus on two notable cases obtained from the P55 and P77-3 samples, as illustrated in Figure 8 and Figure 9.

**Figure 10:**
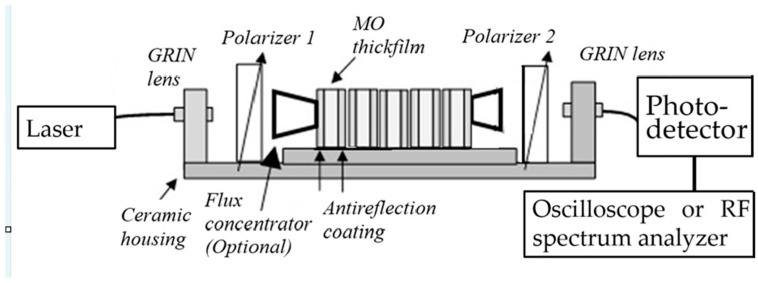
A magneto-optic B-field sensor utilizes MO thick films that are tightly stacked without air gaps to enhance the magnetization proximity effect when exposed to an external magnetic field. The probe beam at 1550 nm is linearly polarized and generated by a highly stable laser. Antireflection coatings on the MO films improve the transmission of the probe beam through the stacked films. The probe beam exiting the second polarizer is amplitude-modulated in proportion to the magnetic field strength. This modulated signal is then converted into an electric signal by a photodetector.

The MO property characterizations were conducted using a thick film sample placed in an MO sensor housing similar to the one shown in Figure 10. In this setup, we installed only a single MO sample in the sensor housing without using flux concentrators. Based on the data presented in Figure 6, it would be optimal to select a probe beam wavelength between 700 and 800 nm to achieve maximum Faraday rotation. However, since a highly stable laser at that wavelength was not available (it needs to maintain extreme stability—both polarization and amplitude—over an extended period), we opted to use a very stable 1550 nm laser as our probe beam.

A linearly polarized laser beam with a wavelength of 1550 nm and a typical power of 17 mW is directed through a polarization-maintaining (PM) fiber, then passes through a thin polarizer and enters an MO-thick film. After exiting the MO sample, the probe beam passes through an analyzer that acts as a cross-polarizer relative to the first polarizer. The MO sample testing housing, containing the thick film, is placed inside a transverse electromagnetic (TEM) cell within a Faraday cage to shield external electromagnetic interference. The TEM cell produces an RF electric field vertically between the septum and the TEM shell, while the magnetic field is perpendicular to it. We have oriented the MO sample inside the sensor housing so that the surface of the thick film is positioned at various angles relative to the RF magnetic field and the probe beam. In Figure 8 and Figure 9, the thick red arrow indicates the direction of the probe beam, while the thick purple arrow shows the direction of the RF magnetic pulse in relation to the magneto-optical thick film sample.

Figure 8 shows one of our data sets: the Faraday rotation (MO response) of a thick-film sample P55. This was measured at various probe-beam angles, while the direction of the magnetic pulse was perpendicular to the sample, with a maximum strength of just over 7 Gauss. When both the magnetic pulse and the probe beam are aligned parallel to the surface normal of the MO thick film (i.e., $\hat{k}||\hat{n}$), the MO signal (blue waveform) closely resembles the waveform of the magnetic pulse (green waveform). This fast response is attributed to the rotation of magnetization within the magnetic domains.

The MO response varies significantly when the probe beam enters the sample at an angle of approximately 45–50°, particularly in configuration (b), where the probe beam travels from the right to the left side at an angle 45–50°, as represented by the black waveform. Interestingly, when the beam approaches from the top to the bottom at a 45° angle (configuration c), the MO response is only slightly slower (red waveform). These different MO responses are related to the magnetic domain structure within the sample. Because the beam is entering the sample at an angle, its path length through the sample is longer compared to normal incidence. We normalized the data by considering the path length and the refractive index of the sample at 1550 nm.

We next analyzed another magneto-optical (MO) film, labeled P77-3. Figure 9 illustrates the MO response for the thick film sample P77-3. Our polarization microscopy revealed narrow and complex meander-line magnetic domains within the sample, as shown in the inset. In configuration (a), when both the probe beam and the magnetic field are oriented at +45° as viewed from the top of the sample, the MO signal (blue waveform) closely resembles the waveform of the magnetic pulse, although it has a significantly reduced amplitude. In contrast, in configuration (b), where the probe beam and the magnetic field are oriented at −45°, the MO signal (black waveform) appears noticeably distorted. This signal has a slower response, which can be attributed to the sluggish movements of the magnetic domains. This distortion indicates domain wall motion, which typically occurs at slower speeds. Interestingly, the amplitude in this configuration is greater than that observed in configuration (a).

The fast and slow MO responses in P77-3 depend on the alignment of the probe beam and the magnetic field directions relative to the hard and easy axes of the magnetic domains. We believe that when the alignment is with the hard axis, it results in magnetization rotation within the domains. Conversely, if the alignment is with the easy axis, the magnetic field induces domain-wall motion, which can be detected by the probe beam. These phenomena are similar to those observed in sample P55.

In contrast to P55, where the domains are larger, the domains in P77-3 are much narrower. This size difference means that the probe beam, which is significantly wider than the domain sizes, interacts with multiple domains simultaneously. In the case of a fast MO response, a significant portion of magnetization rotations across these domains occurs out of phase (incoherently). Consequently, their effects tend to cancel each other out, leading to a reduced amplitude of the MO response. Conversely, in the case of a slow MO response, domain wall movements across the different domains occur in phase, resulting in a larger amplitude of the MO response.

## 4. Ultra-Wideband Magneto-Optic Field Sensor

Considering how magnetic domains affect Faraday rotation in MO thick films, we specifically selected films that have larger magnetic domains, similar domain patterns, and more coherent MO responses. Most of these films were grown in the same batch. These thick films typically range from 300 to 500 µm in thickness, and we stacked them as shown in Figure 10. This stacking increases the path length of the probe beam, significantly enhancing the sensitivity of the MO magnetic field sensor.

**Figure 11:**
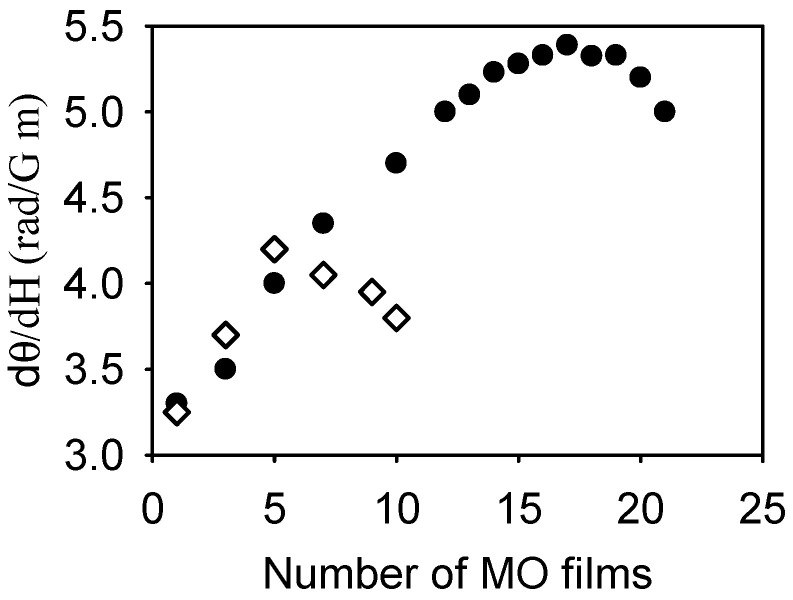
Faraday rotation per magnetic field as a function of the number of stacked MO films. Solid circles represent tightly stacked P55 MO films, and Diamonds indicate P55 MO films that are evenly spaced with approximately a 1 mm gap between them.

We observed that the sensor’s sensitivity increased with the number of stacked MO films, reaching a peak at 16 to 20 films. Beyond this point, the sensitivity began to drop rapidly with the addition of more films. Additionally, the sensor with tightly stacked MO films—where there is no air gap between them—exhibited noticeably higher sensitivity. This proximity effect occurs when the magnetic domains of one MO film influence those of the adjacent film, resulting in assimilated magnetic domains across the stacked MO films. Figure 11 shows such an example. In the figure, solid circles represent tightly stacked P55 MO films, which are arranged to achieve the magnetization proximity effect. Diamonds indicate P55 MO films that are evenly spaced with approximately a 1 mm gap between them. As the number of stacked MO films increases, Faraday rotation increases due to the longer path length of the MO material.

However, as the number of films increases, the intensity of the probe beam is more attenuated, and the beam polarization experiences greater disruption. These two opposing effects compete until beam disruption begins to dominate when more than 16–20 MO films are stacked together, at which point the detected MO signal starts to decrease rapidly. This disruption occurs even earlier when the MO films are evenly spaced with gaps between them [19].

**Figure 12:**
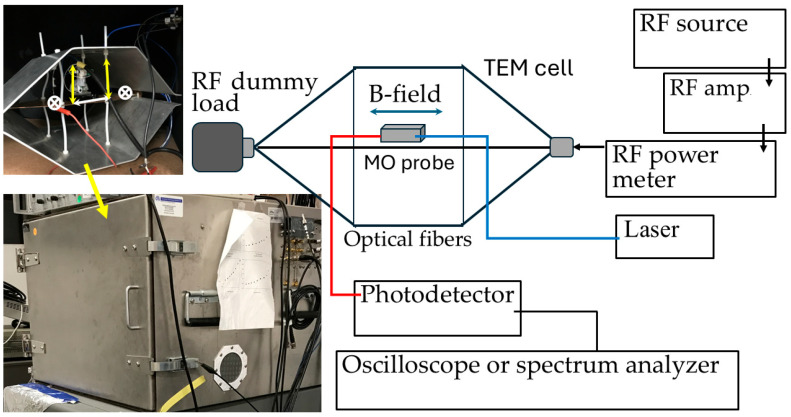
An experimental setup with a TEM cell (shown on the top left) placed inside a Faraday cage (shown on the bottom left) for characterizing an MO sensor. The yellow arrows within the TEM cell indicate the RF E-field, while the white crossed circles and the white arrow represent the RF B-field, which is orthogonal to the E-field. The MO sensor is aligned parallel to the B-field.

**Figure 13:**
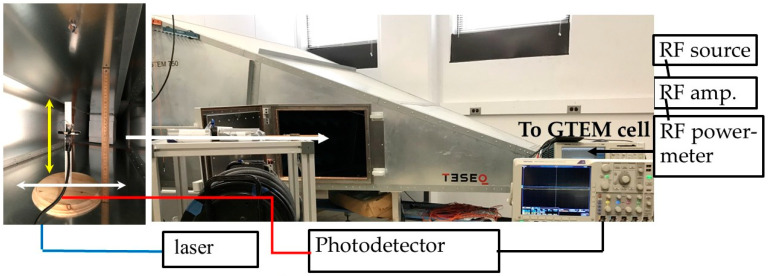
An experimental configuration using a TESEQ 750 GTEM cell to characterize our MO field sensor. The picture on the left shows the MO sensor setup placed inside the GTEM cell. The yellow arrow indicates the direction of the RF electric field. The white arrow indicates the direction of the RF magnetic field inside the GTEM cell, with the MO sensor aligned parallel to it.

We characterized this prototype magnetic field sensor by placing it in one of three test cells: a parallel-plate test cell with dual Helmholtz coils, a transverse electromagnetic (TEM) cell, or a gigahertz transverse electromagnetic (GTEM) cell. To minimize environmental noise during the sensor’s noise-floor test, we enclosed the test setup—either the parallel plate test cell or the TEM cell—within a Faraday cage, except for the GTEM cell test, as shown in Figure 12 and Figure 13.

Our TEM cell, fabricated at the Naval Research Laboratory, can handle frequencies from low frequency to 1 GHz. When an AC or RF signal is fed into the TEM cell, an E-field is produced vertically between the septum and the upper (and the lower) shell, while the magnetic field is produced perpendicular to the E-field. We placed an MO sensor on the septum near the center of the TEM cell and aligned it parallel to the magnetic field. For a low-frequency AC signal, we calibrated the H-field within the parallel-plate test cell, or TEM cell, using a high-precision current meter and Gauss meters (F.W. Bell 5180 and 5170, OECO LLC, Milwaukie, OR, USA). For an RF signal, we calibrated the RF B-field using RF power meters (Agilent E4418B with an E4413A probe, Agilent Technologies, Inc., Santa Clara, CA, USA and the PowerOne power meter from COMM-connect, Raasigvangen 2, Slangerup, Denmark). Since the TEM cell was limited to 1 GHz, we also used a TESEQ 750 GTEM cell (TESEQ GmbH., Berlin, Germany), which, according to its specifications, can produce electromagnetic field signals over the frequency range from DC to 20 GHz. We cross-checked our calibration data by comparing it with NIST calibration data obtained for our electro-optic field sensor, the electric-field counterpart to the MO sensor.

**Figure 14:**
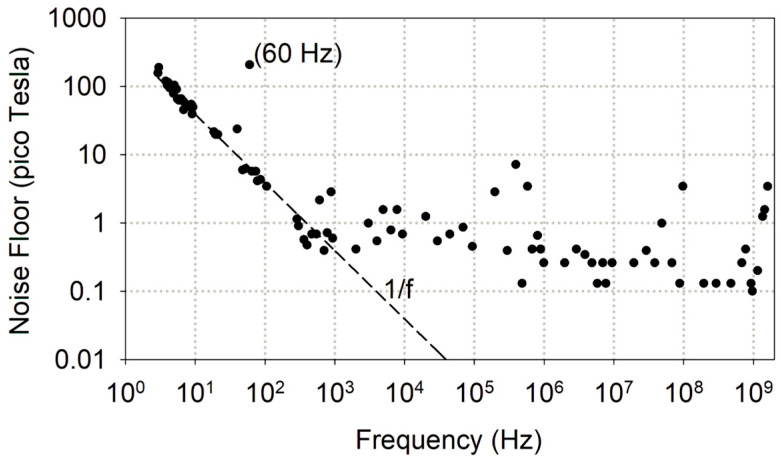
The noise floor of a prototype MO field sensor that we constructed. The data reveal that noise below 1 kHz is primarily influenced by 1/f noise, as represented by the dashed line. The sensor can detect magnetic fields as low as 1 pico-Tesla at frequencies above 1 kHz. Some data points near 60 Hz are attributed to environmental noise, primarily from utility lines, suggesting that the Faraday cage used in the experiment did not provide adequate shielding. The MO sensor’s ability to detect fields deteriorates rapidly at frequencies above 1.3–2 GHz.

As shown in Figure 14, the prototype sensor is capable of detecting magnetic fields as weak as 1 picoTesla within a frequency range of 1 kHz to 1.3–2 GHz. This sensitivity is similar to that of a low-end radio-frequency superconducting quantum interference device (RF-SQUID). The sensor exhibits a relatively flat frequency response above 1 kHz; however, below 1 kHz, there is a significant increase in noise, primarily due to 1/f noise.

We noticed a few outlier data points around 60 Hz, likely caused by environmental noise primarily originating from the utility line. This suggests that the Faraday cage used in our experiment did not provide adequate shielding. Notably, at 2.9 Hz, the sensor’s minimum detectable field can reach approximately 190 pico-Tesla. The estimated error in the measurements, including calibration inaccuracies, typically ranges between 5% and 10% across the entire frequency spectrum.

**Figure 15:**
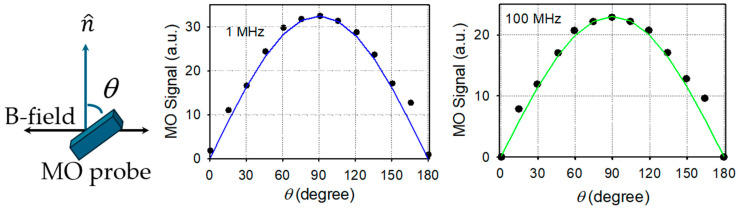
The directional dependence of our magnetic field sensor in relation to the external magnetic field. As illustrated in the diagram on the left, the angle *θ* is measured with respect to the normal of the magnetic field. We present two examples: one at 1 MHz and another at 100 MHz. Our findings indicate a consistent directional dependence across the broad frequency range we studied, which spans from 1 kHz to 1 GHz.

Equations (1) and (2) imply that the MO signal strength of our sensor should display a directional dependence on the magnetic field or magnetization. We examined the strength of the MO signal by varying the orientation of the MO sensor relative to the RF B-field direction, while keeping the RF B-field strength constant, as illustrated in the two examples shown in Figure 15. We confirmed the directional dependence across a wide range of frequencies. While other conventional B-field sensors or antennas may also exhibit some directional dependence, their directional dependence is inconsistent across frequency ranges and is often distorted and unpredictable at different frequencies [9]. In contrast, our MO field sensor demonstrates a highly consistent directional dependence throughout the frequency range, making it suitable for use as a vector field sensor [19].

We also assembled a few other MO field probes with a design similar to that shown in Figure 10, but we used a CdMnTe single crystal instead of the Bi:RIG material. The CdMnTe family exhibits distinctly different magneto-optical properties that depend on their specific chemical compositions; however, we will not elaborate these variations in this paper.

**Figure 16:**
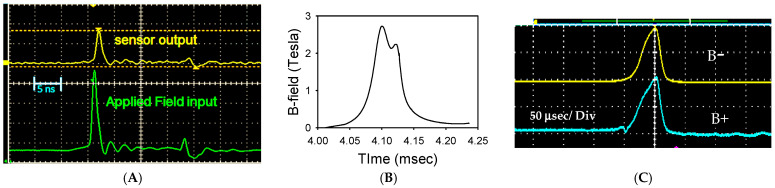
(**A**) High-frequency magnetic field data were collected using a MO field sensor made of Bi:RIG crystals. The magnetic field produced at a high-field test site exceeded a few hundred mT. (**B**) A high-magnetic-field pulse was measured using a MO sensor made of Bi:RIG crystals. (**C**) High magnetic pulses (>60 T) were measured using two CdMnTe MO sensors installed on positive and negative solid wires with extremely high currents (>$3\times 10^{5}$ A) flowing through them.

The sensitivity of magnetic field probes made from CdMnTe crystals is significantly lower than that of Bi:RIG probes. However, CdMnTe probes offer a much wider dynamic range, enabling them to measure magnetic fields from nano-Teslas (nT) up to 120 Tesla. This capability has been demonstrated in experiments conducted at high-field test sites and in other extreme magnetic field tests. Figure 16 presents three examples of our experimental data: one from high-power RF testing and two from high-field experiments, specifically a high-magnetic pulse test and a high-current test.

Our experiments with MO field probes made from Bi:RIG and CdMnTe materials have shown that our MO sensor technology can produce a nearly non-intrusive magnetic field probe. This technology operates over a broad frequency range from sub-Hz to 2 GHz and offers a dynamic range exceeding 100 dB. In addition to our initially intended applications, such as RF and microwave testing and evaluation, we anticipate that this technology will open up a variety of other uses. These include low-frequency magnetic applications and biomedical uses, which benefit from its operation at room temperature, high sensitivity, ultra-wide bandwidth, and exceptional dynamic range.

## 5. Summary

We have developed a prototype for an ultra-wideband magnetic field sensor that utilizes the Faraday effect and the behavior of magnetic domains in thick films of bismuth iron garnet (Bi:RIG). Our magneto-optical field sensor is designed to measure magnetic fields across a very wide frequency range with minimal disturbance to the magnetic field that it measures. It can detect magnetic fields as low as 1 pT within a frequency range of 1 kHz to 1.3–2 GHz, exhibiting a relatively flat frequency response.

At frequencies below 1 kHz, the sensor’s noise floor is influenced by 1/f noise, which results in an increase in the minimum detectable magnetic field as the frequency decreases. For example, at 2.9 Hz, the noise floor of the sensor reaches approximately 190 pT.

However, using the Faraday effect in a ferrimagnetic thick film poses several challenges. The complex behavior of magnetic domains leads to technical difficulties and limits the frequency bandwidth of the magneto-optical response. Further research is needed to address these challenges and to extend the frequency bandwidth of the magneto-optical sensor technology beyond 2 GHz.

## Figures and Tables

**Table 1:** Typical sizes, field perturbation and operating temperatures of magnetometers.

Sensor Technology	Typical Size	Invasiveness	Operating Temperature
Hall Probe	~5 × 3 × 30 mm^3^	Invasive	Room temperature
SQUID	>10^3^ cm^3^ w/cryogenic Dewar	Invasive	Low temp. cryognic
Vapor cell	>A few cm^3^	Invasive	Requires oven for atomic vapor
NRL MO sensor	≤3 × 3 × 20 mm^3^	Nearly noninvasive	Room temperature

## Data Availability

The authors can provide the raw data supporting the conclusions of this article upon request, with the approval of the Naval Research Laboratory.
